# Building a framework for the evaluation of knowledge translation for the Canadian Network for Observational Drug Effect Studies

**DOI:** 10.1002/pds.4738

**Published:** 2019-02-20

**Authors:** Ingrid S. Sketris, Nancy Carter, Robyn L. Traynor, Dorian Watts, Kim Kelly, On behalf of the following contributing members of the CNODES Knowledge Translation Team: Pierre Ernst, JM Gamble, Brenda Hemmelgarn, Colleen Metge, Michael Paterson, Robert Platt W. and Gary Teare

**Affiliations:** ^1^ Faculty of Health Professions, College of Pharmacy Dalhousie University Halifax Canada; ^2^ REAL Evaluation Services Nova Scotia Health Research Foundation Halifax Canada; ^3^ Department of Community Health & Epidemiology Dalhousie University Halifax Canada; ^4^ Nova Scotia Health Authority Halifax Canada

**Keywords:** developmental evaluation, drug safety, knowledge translation, pharmacoepidemiology, research impact

## Abstract

**Purpose:**

The Canadian Network for Observational Drug Effect Studies (CNODES), a network of pharmacoepidemiologists and other researchers from seven provincial sites, provides evidence on the benefits and risks of drugs used by Canadians. The Knowledge Translation Team, one of CNODES' four main teams, evaluates the impact of its efforts using an iterative and emergent approach. This article shares key lessons from early evaluation phases, including identifying stakeholders and their evaluation needs, choosing evaluation theories and approaches, and developing evaluation questions, designs, and methods appropriate for the CNODES context.

**Methods:**

Stakeholder analysis was conducted using documentary analysis to determine key contextual factors and research evidence needs of decision maker partners and other stakeholders. Selected theories and frameworks from the evaluation and knowledge translation literature informed decisions about evaluation design and implementation. A developmental approach to evaluation was deemed appropriate due to the innovative, complex, and ever‐changing context.

**Results:**

A theory of change, logic model, and potential evaluation questions were developed, informed by the stakeholder analysis. Early indicators of program impact (citation metrics, alternative metrics) have been documented; efforts to collect data on additional indicators are ongoing.

**Conclusion:**

A flexible, iterative, and emergent evaluation approach allows the Knowledge Translation Team to apply lessons learned from completed projects to ongoing research projects, adapt its approaches based on stakeholder needs, document successes, and be accountable to funders/stakeholders. This evaluation approach may be useful for other international pharmacoepidemiology research networks planning and implementing evaluations of similarly complex, multistakeholder initiatives that are subject to constant change.

**KEY POINTS**

1. Developmental evaluation is an appropriate approach for evaluating innovative and complex programs, with ever‐changing contexts and key players.
2. The purposes of the Canadian Network for Observational Drug Effect Studies (CNODES)' knowledge translation evaluation are to: (1) be accountable to funders and other stakeholders; (2) demonstrate value in CNODES' knowledge translation activities; and (3) learn about the efficiency, effectiveness, and outcomes of CNODES' knowledge translation activities and inform decisions related to improving and evolving these activities.
3. CNODES' knowledge translation evaluation plan incorporates concepts, theories, and frameworks from the social, organizational, political, and clinical sciences; it includes a nested logic model—to ensure alignment with broader CNODES and Drug Safety and Effectiveness Network (DSEN) evaluation elements—and a theory of change, evaluation questions, and key indicators.
4. A flexible developmental evaluation approach allows CNODES to improve its knowledge translation activities by learning from its ongoing research projects, adapting its approaches, and documenting its research impact.
5. Other networks may adapt and expand on CNODES' approach and lessons learned, including the importance of involving an evaluation expert and understanding key contextual factors and needs of stakeholders.

## 1 INTRODUCTION

### 1.1 Background

Government‐funded research networks are increasingly called upon to document the results of their knowledge translation activities and assess the impact of their research.^1^, ^2^, ^3^, ^4^ The Canadian Network for Observational Drug Effect Studies (CNODES), funded by the Canadian Institutes of Health Research (CIHR), *
The Canadian Institutes of Health Research (CIHR) is the Government of Canada's health research investment agency. CIHR was created in 2000 under the authority of the Canadian Institutes of Health Research Act. It is an independent agency and is accountable to Parliament through the Minister of Health.^5^
 is no exception.^5^ This increased focus on impact assessment is embedded in the Canadian and international research landscapes. Research productivity metrics and other measures of impact are used in many countries, in part, to assess institutions' rankings and then determine the level of institutional research funding received from governments.^1^, ^6^, ^7^, ^8^ However, Canada's federal government does not have a formal national research productivity assessment system of their higher education institutions,^9^ partly because education is a provincial jurisdiction. Nevertheless, funders, government agencies, and institutions are increasingly requiring individual researchers and research groups to provide evidence of the impact of their research, †
For government‐funded research organizations, the need to provide evidence of research impact is part of the broader government accountability framework and 2016 Policy on Results.^10^
 especially in health services research.^11^

Traditionally, research productivity has been assessed using metrics such as number of peer‐reviewed articles, journal impact factor, and number of citations.^9^, ^12^ These metrics, while relatively easy to measure and routinely used in academic research settings, do not always effectively nor sufficiently capture the impacts beyond the academic community on practice and policy in applied research settings.^13^, ^14^, ^15^ Measuring the impact of applied research, such as that produced through CNODES, is complex. Such research networks often face unpredictability, nonlinearity, and unanticipated changes and challenges to predicting cause‐effect relationships.^16^

CNODES ‡
For more information on CNODES' structure and governance, please refer to other articles in this special *Pharmacoepidemiology & Drug Safety* supplement. is a research network of pharmacoepidemiologists and other researchers distributed across seven Canadian provincial sites^17^ that conducts research to aid in understanding the benefits and risks of drugs after they enter the market. De‐identified Canadian population–based administrative health care data are a key data source for this work. Data from the United States and United Kingdom are also included, when required, due to the rarity of some adverse reactions and/or diseases. The data from different sources are pooled using techniques such as meta‐analysis, guided by the literature and key reports, to provide more accurate estimates than individual Canadian provincial research groups.^18^, ^19^ As one of the collaborating centers of CIHR's Drug Safety and Effectiveness Network (DSEN), §
DSEN was funded in 2010 by the CIHR, in collaboration with Health Canada and other stakeholders.^20^
 CNODES also works with other DSEN collaborating centers to respond to queries on drug safety and effectiveness from public sector decision makers and other stakeholders.^20^

Individual CNODES research projects have a high level of technical complexity because they frequently require establishing new data linkages and adapting research methods to meet the needs of stakeholders. Further, the ever‐evolving roster of new researchers and trainees involved in CNODES projects contributes to organizational complexity.

The environment in which CNODES functions is dynamic due to government organizations' changing structures, personnel, priorities, programs, and policies; new drugs coming on and off the market; new drug safety signals; and other drug information being generated both in Canada and internationally.

The CNODES network includes four main teams: Database, Methods, Training, and Knowledge Translation. The CNODES Knowledge Translation Team leads activities that translate and mobilize knowledge from research projects for use by Query Submitters (Health Canada, Federal/Provincial/Territorial (F/P/T) decision makers, and others) where this research is one input to the decision‐making process related to drug safety and effectiveness. Decision makers may also incorporate other potential influences such as public opinion, media reporting of issues, economic climate, policy infrastructure, political ideology and priorities, stakeholder interests, expert advice, industry perspectives, health professional regulatory body standards, prescriber environment and resources, and patient needs, values, and preferences.^3^, ^21^, ^22^ The CNODES Knowledge Translation Team works with CNODES researchers and staff, DSEN, and decision makers to develop and strengthen linkages, trust, respect, and partnerships to facilitate their collective contribution to promote drug safety for Canadians.

In a complex context, such as CNODES, traditional methods and approaches to evaluation may be insufficient.^23^ The purpose of this article is to share our experience and insights in using a developmental approach to evaluate the design, implementation, operations, and effectiveness of CNODES' knowledge translation activities. Inherent in the evaluation theories and frameworks employed in this approach is the consideration of individual‐level human factors that may influence program activities and outcomes.

## 2 CONTEXT

### 2.1 Selected aspects of Canada's drug safety and effectiveness system

#### 2.1.1 Selected legislation and reports

Several recent reports, initiatives, and legislation have led to a strengthening of specific aspects of Canada's drug safety and effectiveness system. A 2008 parliamentary committee report entitled “Post‐Market Surveillance of Pharmaceuticals” recommended the establishment of a drug safety and effectiveness network,^24^ which led to the creation of DSEN in 2010.^20^, ^25^, ^26^, ^27^ Both a Health Council of Canada commissioned article^28^ and a Council of Canadian Academies assessment report^29^ noted the need for more effective drug risk communication. The Health Council of Canada article called for Health Canada to work together with provinces, territories and other government agencies, health care practitioners, and consumers to develop, monitor, and evaluate the effectiveness of drug safety messaging.^28^ The Council of Canadian Academies' report provides a roadmap for risk communication based on the premise that risk characterization, management, and communication is a dynamic, socially and politically interactive process.^29^ A 2015 report on health care innovation^30^ discusses the need for a strong Canadian post‐market safety and effectiveness system.

Vanessa's Law (the Protecting Canadians from Unsafe Drug Act—Bill C‐17, November 2014) amended the Food and Drugs Act and sought to improve the safety of drugs and medical devices, with some legislation ¶
Under this legislation, the Minister can require a person to disclose confidential business information, request modifications to labeling or packaging, or order a recall. Regulations are being developed to enable Health Canada to exercise some of the other authorities in Vanessa's Law.^31^
 coming into force immediately and other regulations under development.^32^

While the origins of the need for further post‐marketing drug safety and effectiveness information go back many decades, the National Pharmaceuticals Strategy, which was established in 2004, had specific proposals to address the concerns about drug effectiveness, safety, and affordability.^33^, ^34^, ^35^, ^36^, ^37^, ^38^ The nine‐element strategy was part of the First Ministers' 10‐year plan to strengthen health care; one of the strategy's elements was to “strengthen evaluation of real‐world drug safety and effectiveness.”^33^, ^34^ Many reports on drug safety and other information led to the announcement by the federal government of the establishment of DSEN at CIHR.

#### 2.1.2 Organizations mandated to improve drug safety and effectiveness for pharmaceutical management

Health Canada is composed of branches, offices, bureaus, and agencies (Appendix S1). As mentioned above, CNODES is a collaborating center of DSEN and funded by CIHR. The Health Products and Food Branch's (HPFB) mandate is to evaluate and manage health‐related benefits and risks of therapeutic products. The branch provides a regulatory framework for therapeutic products such as prescription and nonprescription drugs as well as benefit‐risk assessment information, using a product life cycle approach, and provides information to patients and their health care providers to assist them with decisions related to drug therapy.^39^, ^40^, ^41^ The activities of the HPFB are carried out through a variety of directorates and offices located in the National Capital Region (Ottawa‐Gatineau) and five regional offices (Atlantic, Quebec, Ontario, Manitoba‐Saskatchewan, Western) across the country. Of note for drug safety and effectiveness are the Marketed Health Products Directorate (MHPD), the Office of Pediatric Initiatives (OPI), the Policy, Planning and International Affairs Directorate (PPIAD), the Office of Controlled Substances (OCS), the Strategic Policy Branch, and the Therapeutic Products Directorate (TPD) (Appendix S1).

While Health Canada is responsible for drug product regulation in Canada, individual provinces and territories are responsible for health care program delivery, including most of the public drug insurance programs. #
Canada Health Act (R.S.C., 1985, c. C‐6): http://laws-lois.justice.gc.ca/eng/acts/C-6/. The federal government also provides prescription drug coverage for eligible groups, such as First Nations and Inuit Non‐Insured Health Benefits, veterans, members of the military and Royal Canadian Mounted Police (RCMP), and inmates in federal penitentiaries. The F/P/T pharmacare programs finance and manage pharmaceutical benefits including determining the financing of the program and those individuals eligible for benefits, as well as which drugs to reimburse and under what conditions. These programs examine the impact of specific programs and policies on patients' access to medicines, adherence, health outcomes, and cost.

In a number of situations, Health Canada seeks information and advice from a variety of external sources. There are certain external advisory bodies that outline, in their terms of reference, different ways of advising the department.^42^ For example, the Enhanced Review Capacity Unit was established to provide centralized contracting services to facilitate the appropriate use of expert advice from external sources (eg, drug or disease experts, professional associations, scientific advisory committees or panels, and academic institutions).^43^

Policies related to drug safety and effectiveness rely on global evidence. Health Canada's assessment of drug safety and effectiveness considers data from a variety of countries accessing both published and confidential information.^39^, ^44^, ^45^, ^46^, ^47^, ^48^, ^49^, ^50^, ^51^ Several international initiatives that provide research evidence related to drug safety and effectiveness are described in Table 1. CNODES researchers also connect with researchers from these organizations at conferences and other international meetings, such as the International Society for Pharmacoepidemiology (ISPE) annual conference. **
ISPE's upcoming and previous annual conferences: https://www.pharmacoepi.org/meetings/conference.cfm. CNODES, and many of the other international initiatives described in Table 1, use electronic health data for pharmacoepidemiologic studies and work on developing methodologies to improve drug safety benefit and risk assessments.^70^

**Table 1:** Selected international initiatives that provide research evidence related to drug safety and effectiveness

Initiative	Description
Europe	
Pharmacoepidemiological Research on Outcomes of Therapeutics by a European Consortium (PROTECT)^52^	The overall objective of PROTECT is to strengthen the monitoring of the benefit–risk of medicines in Europe and, as such, it has been designed as a comprehensive and integrated project aiming to develop and validate a set of innovative tools and methods to be used in the field of pharmacoepidemiology and pharmacovigilance.^53^ The European Medicines Agency (EMA) is the coordinator and GlaxoSmithKline (GSK) is the deputy coordinator of PROTECT. They manage a multi‐national consortium of 34 partners including academics, regulators, small‐ and medium‐sized enterprises, and European Federation of Pharmaceutical Industries and Associations (EFPIA) companies.
European Network of Centres for Pharmacoepidemiology and Pharmacovigilance (ENCePP)^54^	ENCePP, initiated in 2007, aims to increase capacity for pharmacoepidemiology research in Europe, define common methodological standards, and propose governance principles for the conduct of collaborative studies. As of July 31, 2017, ENCePP has included 168 centers, who focus on pharmacoepidemiology or pharmacovigilance, from 18 European countries. Through the centers, ENCePP provides access to a large pool of experts who strongly support the operation of the new pharmacovigilance legislation by complementing regulatory guidance with methodological recommendations. The new culture of collaboration, common scientific standards, and common governance principles introduced by ENCePP is suggested to greatly facilitate the establishment of research consortia.
Exploring and Understanding Adverse Drug Reactions by Integrative Mining of Clinical Records and Biomedical Knowledge (EU‐ADR) Alliance^55^	EU‐ADR Alliance is operated by the EMA under Horizon 2020. This is being extended into the European Medical Information Framework.^56^
Scalable, Standard based Interoperability Framework for Sustainable Proactive Post Market Safety Studies (SALUS)^57^	SALUS aims to provide a standard‐based interoperability framework that will enable execution of safety studies for mining and analyzing real‐time patient data in communication with disparate heterogeneous electronic health record systems.
Pharmacovigilance Risk Assessment Committee (PRAC)^58^	PRAC is the EMA's committee responsible for assessing and monitoring the safety of human medicines.
The Integration of Content Management Information on the Territory of Patients with Complex Diseases or with Chronic Conditions (MATRICE)^59^	MATRICE is a national network funded by the Italian Ministry of Health. The network covers a population of about 9 million people living in some of the local health authorities in 9 of the 21 regional health care systems in Italy. The distributed network is used to evaluate the impact of health policies on quality and equity of health care. The network participates in several studies funded by the Italian Ministry of Health.^49^
United States	
Sentinel^60^	Sentinel is funded by the US Food and Drug Administration (FDA) to study the safety of medical products. Sentinel data partners include private insurers, the Center for Medicare and Medicaid Services, the Veterans Health Administration, and the Department of Defense. Sentinel uses a common data model.^61^, ^62^
Observational Medical Outcomes Partnership (OMOP)^63^	OMOP was a public‐private partnership established to inform the appropriate use of observational healthcare databases for studying the effects of medical products. The OMOP Pilot concluded in June 2013 after achieving its mission. The community is actively using the OMOP common data model and vocabulary for their various research purposes. The OMOP lab was transferred to the Reagan‐Udall Foundation (RUF) for the FDA under the IMEDS Program, and has been re‐branded as the IMEDS Lab (see below).^64^
Innovation in Medical Evidence Development and Surveillance (IMEDS)^65^	IMEDS is a program within the RUF. The RUF, a not‐for‐profit organization was authorized through the 2007 FDA Amendments Act to help advance the regulatory science needs of the FDA. It was designed to be a vehicle for bringing an array of resources and perspectives to bear on high priority FDA regulatory science projects. The foundation fosters collaborations between patient groups, industry, academia, and FDA scientists to design and conduct regulatory science research. The IMEDS program is offered by the RUF to help advance the regulatory science needs of FDA. IMEDS is a public‐private partnership created to build upon the significant progress made on research methodology by the Sentinel Initiative, including its Mini‐Sentinel pilot, and OMOP. In mid‐2013, OMOP was transitioned from the Foundation for the National Institutes of Health (FNIH) to the RUF, and OMOP's tools, capabilities, and resources became the foundation for IMEDS' research and operations.
United Kingdom	
Vigilance and Risk Management of Medicines (VRMM) program^66^	VRMM is a division of the Medicines and Healthcare Products Regulatory Agency involved in monitoring drug safety and effectiveness.
Drug Safety Research Unit (DSRU)^67^	DSRU is an independent academic unit which conducts post‐marketing surveillance and pharmacoepidemiology studies in both the UK and Europe.
Multinational	
The International Council for Harmonisation of Technical Requirements for Pharmaceuticals for Human Use^68^	ICH consists of pharmaceutical industry and drug regulators from Europe, Japan, and the US. Their primary area of work is guideline development with the intention of improving the consistency and timeliness of safety reporting for marketed drugs.
International Coalition of Medicines Regulatory Authorities (ICMRA)^69^	ICMRA is a voluntary, executive‐level, strategic coordinating, advocacy, and leadership entity of regulatory authorities that work together to: •address current and emerging human medicine regulatory and safety challenges globally, strategically and in an ongoing, transparent, authoritative, and institutional manner; •provide direction for areas and activities common to many regulatory authorities' missions; •identify areas for potential synergies; and •wherever possible, leverage existing initiatives/enablers and resources.

### 2.2 CNODES' knowledge translation activities

CNODES' knowledge translation activities are designed to ensure CNODES' rigorous and innovative research results are accessible to the Query Submitters (Health Canada, F/P/T decision makers who generated the query) and to assist in providing these results to other decision makers, helping to promote their uptake in the decision‐making process. The goal of CNODES' knowledge translation activities is to provide decision makers—Health Canada and F/P/T health systems, including their drug plans—with research evidence to support the latter's assessment of marketed drug products and, ultimately, the appropriate selection and safe use of drugs.^71^ CNODES' research could help decision makers (1) prioritize issues and understand their causes; (2) develop policy; and/or (3) assess the impact of the chosen policy option.

CNODES develops its knowledge translation approach by incorporating concepts, theories, and frameworks from the social, organizational, political, and clinical sciences.^72^, ^73^, ^74^, ^75^, ^76^, ^77^, ^78^, ^79^, ^80^, ^81^, ^82^ It reviews the empirical evidence to help identify facilitators and barriers to knowledge translation, as well as to determine best approaches for providing researchevidence to decision makers in an accessible manner.^83^, ^84^, ^85^ In their guidance on behavior change, the United Kingdom's National Institute for Health and Care Excellence suggests that implementation needs to take place at multiple levels—individual, organizational, community, and population—for scientific evidence to impact human behavior.^86^ CNODES' Knowledge Translation Team taps the expertise of several organizations affiliated with DSEN and uses DSEN‐established mechanisms to assist with connecting CNODES researchers to decision maker partners. These organizations include the DSEN CIHR/Health Canada Working Group (which generates queries ††
For more information on the DSEN query submission process, refer to the Guidance Document for Submitters of DSEN Queries: http://www.cihr-irsc.gc.ca/e/45932.html.^87^
), the DSEN Science Advisory Committee, and the Canadian Agency for Drugs and Technologies in Health (CADTH).^88^ In addition to its research results, CNODES also shares its innovative methods with researchers and decision makers. CNODES' approach to knowledge translation is influenced by the Knowledge to Action cycle^73^ and uses both end of grant and integrated approaches to translate knowledge, as described below.^89^

#### 2.2.1 CNODES' end of grant knowledge translation

CNODES' end of grant knowledge translation efforts aim to “increase the evidence on drug safety and effectiveness available to regulators, policy‐makers, health care providers, and patients.”^20^ Information on long‐term drug safety and effectiveness may be of direct relevance to clinical and policy decision making at all levels of health care systems within Canada and internationally. CNODES targets actionable messages to its decision maker Query Submitters and other relevant stakeholders. In addition, it shares its innovative methods with other researchers and analysts. CNODES' approach includes traditional academic publications and reports and more intensive knowledge translation strategies (eg, plain language summaries, media releases, targeted presentations), when appropriate and feasible. CNODES' Knowledge Translation Team also draws on trusted sources (ie, individuals and organizations regarded as being reliable, credible messengers for the intended audience), with an extensive user followership, to aid in its knowledge translation efforts. CNODES shares information about its capabilities and accomplishments through promotional materials and, at times, directly disseminates specific project results to relevant organizations. For example, results of the CNODES isotretinoin study, published in April 2016, were shared directly with Health Canada prior to publication. This publication contributed to a “recalls and safety” alert, published in September 2016, which underscored the importance of preventing pregnancy while taking isotretinoin.^90^

#### 2.2.2 CNODES' integrated knowledge translation

CNODES has also more recently adopted an approach for integrated knowledge translation. With this approach, researchers involve knowledge users throughout the research process, from defining the research question and methods to interpreting and disseminating the results.^20^, ^91^ CNODES employs a user‐centric approach that responds to queries ‡‡
Queries can be related to any of the over 13 000 prescription and nonprescription drugs on the Canadian market^92^ and related to different age populations (pediatrics, geriatrics) or disease conditions. from a diverse set of decision makers. §§
Decision makers may include Health Canada, the F/P/T public sector drug plans, or other public sector organizations involved with supporting drug therapy decision making, such as CADTH and the Institut national d'excellence en santé et en services sociaux (INESSS). Other organizations affiliated with provincial health departments, such as Health Quality Councils, can also provide input into queries through their provincial representatives.^87^
 CNODES works directly with Query Submitters to clarify and determine the feasibility of specific research questions.^87^ CNODES also interacts with both the DSEN Coordinating Office and CADTH to translate its research findings to meet the needs of the Query Submitters. Integrated knowledge translation is by nature ever‐evolving as it is intended to be responsive to the needs of stakeholders; a developmental evaluation approach, as discussed below, is well suited to an evolving context and is also best implemented as an integrated team function.^23^

CNODES has been able to support the activities of specific Health Canada directorates (Appendix S1) by providing research results on drug safety and effectiveness to Health Canada directly, as well as communicating the research results to health care professionals and the public through peer‐reviewed articles and the media. Evidence from the CNODES studies involving isotretinoin^93^ and incretins^94^ has been reported separately in Health Canada's MHPD publications.^90^, ^95^ CNODES has also undertaken queries from F/P/T drug plan managers to compare patterns of use and adherence to clinical guidelines across provinces. For example, CNODES has provided evidence in response to a query related to opioids that described changes in dispensing opioid medications following the introduction of a tamper‐deterrent formulation.^96^, ^97^ In another example, CNODES is currently conducting research in response to Health Canada queries related to the appropriate use of fluoroquinolones and the selection of initial antimicrobial therapy in specific disease conditions.

Through its international connections, CNODES is collaborating with the US Food and Drug Administration (FDA) Sentinel Initiative team^48^ to adapt their Common Data Model for use in Canada, which could lead to collaborative studies involving US and Canadian data.

## 3 EVALUATING CNODES' KNOWLEDGE TRANSLATION ACTIVITIES

### 3.1 Evaluation purpose and approach

Consideration of specific methods and indicators for evaluation began early in the establishment of CNODES. ¶¶
Dr Nancy Carter and Dr Anatoliy Gruzd presented at the CNODES semi‐annual meeting held in Halifax, Nova Scotia, in 2013. In addition to ongoing evaluation of the overall CNODES program, it was important to develop an evaluation framework specific to the CNODES knowledge translation component that could be integrated within the evaluation work of the larger program. ##
CNODES intends to expand its evaluation approach to its other teams and broader network goals, as time and resources permit. CNODES has first focused on its operational challenges, which are not insignificant in a network of over 60 researchers. In its first 5 years of operation, CNODES has completed 56 queries for a total of 16 studies, each of which may be repeated in up to nine sites with their specific health databases. The DSEN Coordinating Office was engaged as an evaluation stakeholder to ensure alignment of the CNODES knowledge translation evaluation with DSEN's general evaluation approach and logic model.

The knowledge translation needs of multiple, diverse Query Submitters is, by nature, highly variable and evolving in response to contextual factors such as timelines, resources, and political climate. Knowledge translation activities must also evolve to meet these needs. An evaluation of knowledge translation efforts was deemed necessary for continuous learning and improvement of the knowledge translation process, to demonstrate the role of knowledge translation in the broader CNODES program, and for accountability to stakeholders. Therefore, the purpose of the CNODES knowledge translation evaluation is threefold: (1) for *accountability* to funders and other stakeholders; (2) to *demonstrate value* of CNODES' knowledge translation activities; and (3) to *learn* about the efficiency, effectiveness, and outcomes of CNODES' knowledge translation activities and *inform decisions* related to improving and evolving CNODES knowledge translation activities. Given the complex environment, its multiple stakeholders, their diverse needs, and the continually evolving nature of CNODES' knowledge translation work, expertise in evaluation was sought to provide guidance and support in integrating evaluation throughout all aspects of CNODES' knowledge translation program development, implementation, and refinement.

### 3.2 Theories and frameworks employed

CNODES' knowledge translation evaluation design incorporated elements of several approaches and theories of evaluation including developmental, utilization‐focused, and program theory‐driven evaluation. ***
Specifically included were the International School on Research Impact Assessment (ISRIA) Research Impact Assessment Framework,^98^ the Canadian Academy of Health Sciences (CAHS) framework,^1^ the CIHR Performance Measurement Regime Toolbox,^99^ and various theoretical approaches (eg, contribution analysis and developmental evaluation).^23^, ^100^
 A developmental evaluation approach was chosen because of its usefulness in evaluating complex programs that exist within an environment of constant growth and change. A utilization‐focused approach, a key principle of developmental evaluation, also takes into consideration the diverse perspectives and intended uses of the evaluation by stakeholders and external audiences.^23^, ^101^ CNODES' stakeholders are diverse, as are their expectations of evaluation and the way they use evaluation products. Identifying and acknowledging the diversity of needs and perspectives of stakeholders enables a more comprehensive approach to evaluating CNODES' knowledge translation that will assist in producing evaluation findings that are of high utility to diverse stakeholder groups.

Program theory describes what a program or organization does (ie, activities) and what it hopes to achieve through its actions (ie, outcomes/impact). Theory‐driven evaluation refers to an evaluation that is grounded in program theory.^101^, ^102^, ^103^, ^104^ Program theory is often illustrated using logic models and theories of change. Logic models are graphic representations that connect program activities to the intended outcomes, often using boxes and arrows to show connections. Theories of change build upon logic models by articulating the assumptions associated with carrying out the work and explaining how outcomes will be achieved.^23^, ^79^, ^80^, ^101^, ^104^, ^105^, ^106^, ^107^, ^108^, ^109^ This allows human factors such as beliefs, past experiences, and knowledge, as well as other contextual factors, to be reflected in the theory of change model.^104^ Models may be used, in part, to clarify complex relationships among a program's various components, organize information, provide a common language for all involved stakeholders, and facilitate improved program design, planning, and management.^104^ Multiple reports have noted that the improvement of a medication's benefits/risk balance is complex in a multifaceted health care system; improvement needs attention at the culture and practice levels of health care delivery organizations, the involvement of numerous stakeholders, and strong leadership.^110^, ^111^, ^112^, ^113^

As noted previously, it was important that the CNODES knowledge translation evaluation be compatible with evaluation of other elements of CNODES and DSEN. CNODES has a logic model that includes CNODES knowledge translation as one of several components; this CNODES‐level model was used as the basis for developing a logic model specifically for the CNODES knowledge translation activities. A nested logic model^114^ was used to ensure alignment with evaluation of the broader CNODES program. A nested logic model includes multiple logic models developed at various levels of the initiative, each “nesting” into one another and connecting through shared outcomes. The highest level of a nested logic model is concise, limited in details, and is of greatest use for explaining basic program structure and overarching goals to external stakeholders. Each component of the high‐level logic model is further broken down into a separate logic model that details specific activities, outputs, and outcomes. In the case of CNODES, logic models were developed for each component part including drug safety and effectiveness question development, the research process, capacity building, and knowledge translation. Compartmentalization of structural complexity into multiple models allows the logic model to be continually updated to reflect dynamic changes in components of the broader program.^16^ Applying both a developmental and a nested approach has allowed the CNODES Knowledge Translation Team to systematically design an evaluation plan that is clearly aligned with the intended outcomes and system level impacts of both CNODES and DSEN but flexible enough to reflect the evolving needs of Query Submitters.

A nested approach allows for multiple theories of change to be established when a single theory of change is not sufficient for capturing the complexity of an initiative. For example, all components of CNODES are intended to contribute to improvements in decision‐making, assessment of drug benefits and risks, and building a strong post‐market observational drug research environment; however, the way each element of CNODES contributes to these outcomes may be quite different and not easily expressed using a single theory of change.

In developing the theory of change for CNODES' knowledge translation and considering the relevant assumptions for achieving intended outcomes, it became clear that the impact of the knowledge translation component is contingent, in part, on factors beyond the control of the Knowledge Translation Team and, in some cases, beyond the control of CNODES and its immediate stakeholders. These include, for example, individual‐level factors such as motivation to engage in knowledge translation and opportunities to apply knowledge in practice, organizational‐level factors such as norms for accessing and using evidence to inform decision making, and system‐level factors such as mechanisms and resources for communication among diverse and varied stakeholders (eg, decision makers, researchers, patients, and service providers). This is useful for understanding barriers to achieving outcomes and the best approach for addressing them—for example, whether challenges can be addressed by the Knowledge Translation Team alone or if other teams or stakeholders should be engaged. The theory of change model and logic model †††
Theory of change and logic model were created using DoView Pro for Windows Version 4.0. were also used to help establish evaluation questions and appropriate methods for assessing each stage of implementing CNODES' knowledge translation activities.

### 3.3 Evaluation questions

One challenge faced by many program managers is the demand for reporting on outcomes before such outcomes can feasibly be observed and measured.^115^ Funders and other stakeholders require evidence that a program is making progress toward achieving outcomes, but in many cases, outcomes may not be achieved or measurable for several years. The CNODES knowledge translation evaluation framework outlines questions that are appropriate and feasible for guiding evaluation at each phase of development, implementation, and operation. The evaluation framework established questions based on program theory as defined in the logic model and theory of change and the evaluation needs of stakeholders. By developing evaluation questions for each stage of the program, the reporting requirements can be met in a way that is iterative and satisfies stakeholders' needs. As a result, evaluation activities take place at each stage of implementing CNODES' knowledge translation activities rather than only after all the work of the CNODES Knowledge Translation Team is complete.

By connecting the evaluation questions to the program theory, the CNODES Knowledge Translation Team can demonstrate progress toward intended outcomes and monitor and explain changes in processes and operations. Further, this approach facilitates continuous improvement as the CNODES Knowledge Translation Team can identify and address challenges and barriers to implementation as they arise, thereby increasing the likelihood that established outcomes can be achieved. The iterative nature of a developmental approach means that evaluation questions, indicators, measures, and methods build upon each other and contribute to identifying and addressing challenges (such as limited budgets, time, human resources, and data). In this way, the evaluation and the program are both continuously improving and evolving by adapting to unanticipated changes; not only can challenges be addressed but opportunities for improved methods of data collection, analysis, knowledge translation, and evaluation can also be leveraged.

### 3.4 Stakeholder analysis

The CNODES Knowledge Translation Team conducted an analysis of selected key stakeholders, primarily using documentary review, to better understand the context within which CNODES operates. There are many stakeholders who may be interested in CNODES research, including CIHR, Health Canada, F/P/T pharmacare programs, government organizations, public sector and health care professionals, national and international researchers and trainees, industry, media, voluntary health sector, and patient groups (Appendix S2). Health Canada and F/P/T pharmacare programs have their own roles in setting regulations and policies and delivering programs to provide safe, effective, and affordable drugs. ‡‡‡
Canada Health Act (R.S.C., 1985, c. C‐6): http://laws-lois.justice.gc.ca/eng/acts/C-6/. Their actions set the context in which health care practitioners prescribe, dispense, and monitor drug therapy. The intent of the federal and provincial regulations, policies, and programs includes providing benefit‐risk analysis to inform decision making by health care providers and patients.^39^, ^116^, ^117^ Health authorities may use this information to set organizational drug programs and policies. Patients may use drug safety and effectiveness information provided by health care professionals and others to decide whether and how to take prescription drugs and monitor their effects. The pharmaceutical industry may use the research to contribute to their understanding of the use and effects of their products.

### 3.5 Indicator development

Indicators and measures are being established for evaluation questions related to program implementation, delivery, outcomes, and impact (Table 2). CNODES' Knowledge Translation Team has drafted indicators §§§
Indicators drafted during a Knowledge Translation Team meeting at a CNODES semi‐annual meeting, Winnipeg, May 6, 2015. for its knowledge translation activities and is currently designing rubrics for evaluation. The framework includes an evaluation matrix that connects multiple indicators to each evaluation question, along with sources and methods for collecting data on those indicators. The matrix, like other elements of the evaluation framework, is considered a living document with the opportunity to be refined to adapt to changes in the program and availability of resources, as well as the need for evidence. Consistent with an emergent developmental approach, as the program adapts to changes, the evaluation also adapts, thus remaining useful and relevant.^23^

**Table 2:** CNODES' next 5 years: Objectives, outcomes, performance indicators, and evaluation approach

Objective	Outcomes	Performance indicators	Evaluation approach
1. Increase the reach and diversity of audiences and the customization of knowledge translation approaches.	Short‐term: •increased ability to reach a broad audience; •increased knowledge of the knowledge translation needs of diverse audiences; and •increased ability to tailor knowledge translation to specific audiences.	•#/type of audiences identified; •breadth and depth of audience; •#/type of strategies and products identified and tailored (including French/English translations) for reaching audiences; •#/type of education sessions/products; and •#/type of new and previously existing communication channels.	•document review; •record keeping; •citation metrics/altmetrics; •surveys; and •interviews.
2. Expand on the “pilots” related to a more integrated knowledge translation approach.	Intermediate: •increased use of knowledge translation products; •improved query submission; and •improved communication between researchers and Query Submitters.	•#/type of new and previously existing communication channels; and •#/type of interactions with Query Submitters.	•document review; •record keeping; •surveys; and •interviews.
3. Conduct a stakeholder analysis to understand the facilitators and barriers for primary decision makers.	Short‐term: •increased knowledge of the knowledge translation needs of Query Submitters and potential Query Submitters. Ultimate: •increased ability to meet decision makers' needs.	•#/type of stakeholders consulted; • barriers and facilitators identified; and • solutions and suggestions to overcoming barriers identified and applied.	•surveys; and •interviews.
4. Build a platform and approaches for collaborative multidirectional learning/shared learning/co‐learning with Query Submitters/policy makers, clinicians, and researchers.	Intermediate: •improved query relevance; •improved communication between researchers and Query Submitters; and •improved knowledge translation.	•# requests for knowledge products (pull strategy); •#/types of new and previously existing communication channels; and •#/type of interactions with Query Submitters.	•document review; •record keeping; •surveys; and •interviews.
5. Increase researchers' and trainees' competence, capability, and capacity to engage in knowledge mobilization.	Short‐term: •increased researcher and trainee ability to provide useful knowledge products; and •increased knowledge of the knowledge translation needs of diverse audiences.	•# of educational sessions/products; and •#/type of strategies and products identified and tailored (including French/English translations) for reaching audiences.	•document review; •record keeping; •surveys; and •interviews.
6. Strengthen partnerships with the federal and provincial governments and Health Canada (macro level), and develop partnerships with the health care delivery system (meso level), health care providers, and patients/families and the organizations that represent them (micro level).	Long‐term: •increased knowledge of post‐market drug safety and effectiveness to inform decisions.	•# and provincial distribution of potential champions/knowledge brokers identified; and •# of calls for researchers to brief decision makers.	•document review; •record keeping; •surveys; and •interviews.
7. Measure impact of knowledge translation activities, including conducting a formal social network analysis.	Intermediate: •improved communication between researchers and Query Submitters.	•#/type of new and previously existing communication channels; •#/type of interactions with Query Submitters; •improved understanding of the type and nature of interactions between Query Submitters and researcher; and •strengthening of ties.	•social network analysis; •surveys; and •interviews.

**Figure 1:**
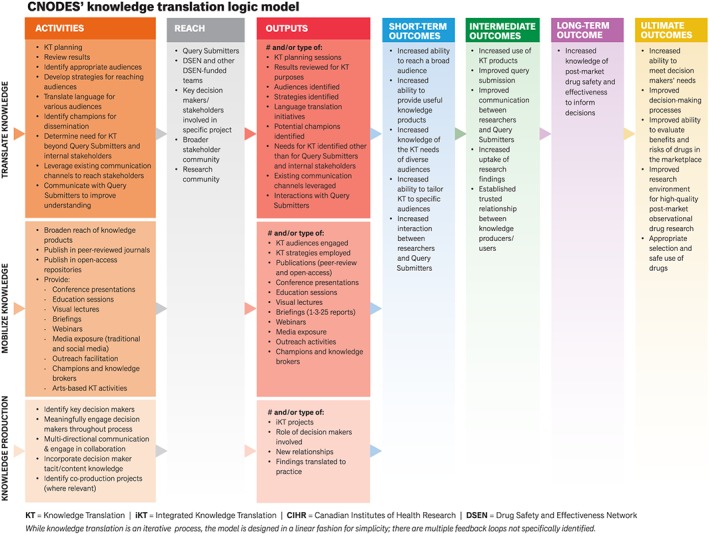
CNODES' knowledge translation logic model [Colour figure can be viewed at http://wileyonlinelibrary.com]

Indicators for implementation and delivery and early outcomes of CNODES' knowledge translation activities were relatively straightforward to identify and track. Many of these indicators are represented by outputs in the CNODES knowledge translation logic model (Figure 1) and are being captured through a combination of citation metrics and altmetrics. Citation metrics are used to track the uptake of published research articles within the academic community.^118^ Altmetrics represent a complementary approach to understanding the reach of CNODES across traditional and social media and to identifying relevant receptor communities (Appendix S3).^119^, ^120^, ^121^ Data on these process and early outcome indicators are being collected selectively for certain articles as part of CNODES' ongoing data collection and monitoring. Establishing indicators and collecting data on longer term outcomes and broader impacts are less straightforward and require multiple indicators and methods to maximize value for a variety of stakeholders.

Currently, several reputable and relevant evaluation frameworks (eg, Canadian Academy of Health Sciences [CAHS] framework,^1^ International School on Research Impact Assessment [ISRIA] framework,^98^ CIHR indicator toolkit,^99^ DSEN logic model^71^) and CNODES' knowledge translation evaluation framework are being consulted by the CNODES Knowledge Translation Team to determine a list of potential indicators that would be useful for reporting on outcomes and impacts. As part of this work, it is necessary to consider not only the indicator but also the resources and expertise required for data collection and analysis of these indicators. Reporting on outcomes will require both quantitative and qualitative data and a mixed methods analytic approach, which is currently under development. The following section summarizes the findings from evaluating CNODES' knowledge translation development, implementation, and early outcomes.

## 4 LESSONS LEARNED FROM DESIGN AND EARLY IMPLEMENTATION OF THE CNODES KNOWLEDGE TRANSLATION EVALUATION FRAMEWORK

To date, using a developmental approach, CNODES' knowledge translation evaluation activities have spanned several types of evaluation—depending on the specific element under scrutiny—including consideration of the appropriateness of the model (proof of concept evaluation), operations (process evaluation), and whether objectives are being achieved as intended (outcome evaluation).^122^ The results of each type are discussed below.

### 4.1 Proof of concept evaluation

Proof of concept evaluation assesses “the rationale for the model, the key characteristics of the model and the organizational structure of the model.”122(p42) To answer the proof of concept questions posed by CNODES (Text Box 1), CNODES' Knowledge Translation Team developed a logic model and theory of change to help connect the work of the Knowledge Translation Team to the broader initiative and to identify assumptions and risks to achieving intended outcomes. Developing a nested logic model and theory of change allowed assessment of the initiative's rationale through articulating CNODES' knowledge translation program theory. It is important to note the logic model and theory of change not only serve to help respond to proof of concept questions but also provide the structure on which the evaluation is built.

The logic model in Figure 1 demonstrates how the Knowledge Translation Team is intended to function by identifying activities and connecting them to outcomes. It is expected that if knowledge is produced, translated, mobilized, and users are engaged (activities completed), this will contribute to reaching a broad audience, providing useful products, tailoring knowledge translation to users' needs, and increasing interaction between researchers and Query Submitters (ie, short‐term outcomes achieved). The achievement of these short‐term outcomes is expected to increase use of knowledge translation products, improve communication between researchers and Query Submitters, improve relationships and uptake of findings, and contribute to increased knowledge of post‐market drug safety and effectiveness to inform decision making (ie, intermediate and long‐term outcomes achieved). The intended ultimate outcome of the broader CNODES program is to contribute to appropriate prescribing, monitoring, dispensing, and use of drugs, and safer drug use systems for Canadians.

**Figure 2:**
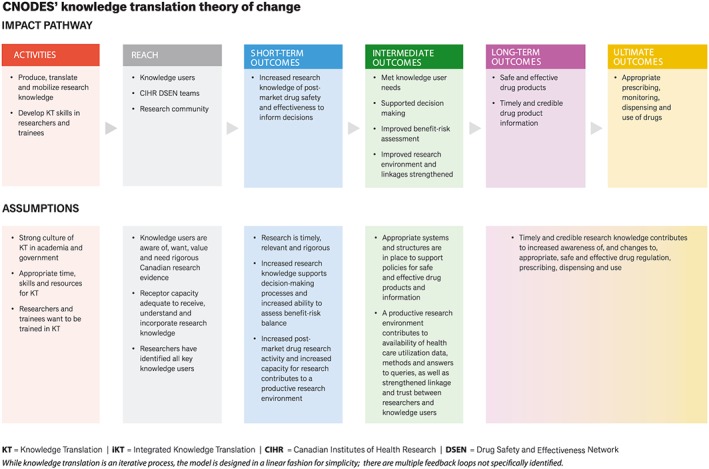
CNODES knowledge translation theory of change [Colour figure can be viewed at http://wileyonlinelibrary.com]

As noted, a nested approach to the logic model helps ensure the Knowledge Translation Team's activities are consistent with and connected to the broader objectives of CNODES and DSEN. Developing a theory of change facilitated a discussion of assumptions that help to realize the impact of the research project and to consider how the project may be impacted if these assumptions are found to be false. For example, researchers—especially early career researchers—may find it difficult to prioritize knowledge translation due to a real or perceived lack of institutional priority for knowledge translation in tenure and promotion guidelines, career progression trajectories, or granting environments. Researchers may also lack formal training and skills in knowledge translation.^123^ In this manner, the theory of change can be used to identify potential barriers to be addressed and potential opportunities to be leveraged. Moreover, the theory of change model is beneficial as a communication tool to demonstrate the requirements for project impact in this complex environment. The theory of change presented in Figure 2 includes a simplified version of the CNODES knowledge translation logic model that is presented in Figure 1 and assumptions associated with each step in the logic model.

As detailed in Figure 2, there are many assumptions about the CNODES context that will impact the extent to which the CNODES work will contribute to the ultimate outcome. The majority of the assumptions are dependent upon aspects of the complex systems in which CNODES is situated. Broadly, there is an overarching assumption that decision makers need further evidence from rigorous Canadian observational pharmacoepidemiologic research to support regulatory and F/P/T drug program decision making. More specifically, a key assumption on which the work of CNODES' knowledge translation is built is that there is a strong culture of knowledge translation in academia and government. If knowledge translation is not highly valued, incentivized, or resourced in either academic or decision maker organizations, there may be challenges in implementing CNODES' knowledge translation activities. This would impact the extent to which outcomes could be achieved. Another assumption is that when knowledge is produced and mobilized, there is receptor capacity to understand and use that knowledge. If the receptor capacity of intended users (eg, pharmacare managers) is limited (eg, due to limited technical and administrative resources or policy constraints), the impact of CNODES' knowledge translation work may also be limited. Assumptions about the broader health system and public sector environments (eg, that supportive cultures and systems, computerized decision support systems, and integrated care across organizations are in place for policies for safe and effective drug products and information) are integral to the ultimate outcomes of appropriate prescribing, dispensing, monitoring, and use of drugs for Canadians.^124^ Each of the assumptions identified in Figure 2 provides important considerations directly related to the potential impact of CNODES.

### 4.2 Process evaluation

Process evaluation considers if an initiative is being delivered as intended (Text Box 1).^122^ Monitoring of CNODES' knowledge translation activities is being used to assess operational efficiency and effectiveness. The CNODES Knowledge Translation Team meets regularly to discuss administrative and operational challenges. The team lead, Dr Ingrid Sketris, also reports on knowledge translation activities regularly to the broader CNODES Steering Committee.

One example of how the CNODES Knowledge Translation Team is conducting their work as intended is how they are engaging with stakeholders. There were many interactions between Health Canada and CNODES prior to publication of the article describing the adherence of isotretinoin users to the pregnancy prevention program.^93^ CNODES presented preliminary results of the isotretinoin safety study to the Health Canada Query Submitters in 2013, focusing on the methods that would be used for assessing pregnancy outcomes and preliminary results related to oral contraceptive dispensations for women on isotretinoin therapy. Health Canada provided feedback which informed the next stages of analysis. In 2014, CNODES conducted additional analyses, added data from the province of British Columbia, and presented updated results to the Health Canada Query Submitters, which were later included in the published article^93^ and subsequent Health Canada publications.^90^, ^125^

### 4.3 Outcome evaluation

Outcome evaluation is used to determine whether objectives are being achieved.^122^ Due to the complex nature of CNODES' knowledge translation environment, outcomes of relevance are somewhat emergent: it is challenging to anticipate the impact of knowledge translation until it happens (Text Box 1). CNODES' Knowledge Translation Team has begun measuring the uptake of its research as an indicator of its reach, use, and impact. For example, in early 2015, CNODES presented the comprehensive analysis and results of the isotretinoin study to Health Canada and finalized the manuscript for submission to a peer‐reviewed journal. In January 2016, CNODES informed Health Canada that the manuscript had been accepted by the Canadian Medical Association Journal and the CNODES Knowledge Translation Team would be drafting a media release. Prior to publication, Health Canada had an opportunity to provide contextual information about the isotretinoin pregnancy prevention program which resulted in modifications of both documents. The article was published in August 2016^93^ and subsequently discussed in Health Canada's Med Effect newsletter in October 2016.^126^ Two CNODES researchers (R.P. and B.W.) were available by teleconference to provide comments on the methods used in this article at the “Scientific Advisory Panel on Isotretinoin Risk Management,” convened on November 17, 2017.^125^

This Panel recommended using both outcome and process measures. Outcome measures included “pregnancy rate (including birth, induced abortion, miscarriage) in sexually active women who are using isotretinoin compared to sexually active age‐matched women who are NOT using isotretinoin, neither of them wishing to become pregnant within the next year” and “… the proportion of newborns with abnormalities ….”

Process measures included “professional adherence (including prescribers and pharmacists) to special requirements before prescribing/dispensing: e.g., discussion of teratogenic risks, two pregnancy tests before starting isotretinoin, counselling on use of two methods of contraception during use and one month after, starting isotretinoin during a menstrual period, monthly negative pregnancy tests prior to isotretinoin refills” and “patient's adherence including contraceptive methods during use and one month after isotretinoin, two pregnancy tests before starting isotretinoin, monthly pregnancy testing.”^125^

The report cited the CNODES study^93^ and provided an extrapolation for the annual pregnancy rate of 16 to 24 per 1000 female users of isotretinoin. The report noted that this rate represents about a 50% decrease in the rate of unintended pregnancy for females aged 15 to 44 years not taking isotretinoin. The panel recommended further monitoring of all contraceptive strategies in this population as well as additional risk mitigation strategies to prevent pregnancy.^125^

The uptake of CNODES' research also extends beyond Canada's borders, in part, with presentations at international meetings,^127^, ^128^ suggesting that CNODES' reach and impact extends beyond its primary goal of providing information to Canadian policy makers, practitioners, and patients. There are several examples to date of how the CNODES' Knowledge Translation Team has contributed to knowledge production through evaluation. The CNODES Knowledge Translation Team has measured its reach through citation metrics^118^ and altmetrics.^119^, ^120^, ^121^ The impact of CNODES' research will continue to be measured through ongoing evaluation processes.

## 5 DISCUSSION

The use of a developmental evaluation approach, including a nested theory of change and logic model, will allow for better communication with the CNODES Steering Committee and with other stakeholders about the work, achievements, and lessons learned by the CNODES Knowledge Translation Team. The evaluation approach provides a mechanism to document implementation of planned activities and the achievement of expected outputs and outcomes, as well as a theory of change to help understand what factors may have influenced the achievement of intended results.^104^ The evaluation methods have helped to elucidate which CNODES knowledge translation activities for specific queries were effective, what circumstances contributed to this effectiveness, and how CNODES' knowledge translation can continue to evolve.

The development and application of the evaluation approach required technical expertise and resources to apply the framework in the CNODES context, whereas indicator development and data collection remain in early stages. This comprehensive evaluation approach adds to more commonly known research impact tools, particularly for those researchers who may have only been exposed to traditional assessment approaches (eg, publishing in high‐impact scholarly journals).

While the context for CNODES is specific in that it is funded by CIHR and provides research evidence to both Health Canada and subnational provincial health systems, pharmacoepidemiologic researchers in other countries may adapt and build upon CNODES' evaluation methods and approach. The impetus for further evaluation work may come from CIHR—where the annual reporting tool for CIHR provides the opportunity to document non‐traditional knowledge translation outputs and outcomes—as well as from Health Canada.

### 5.1 Strengths

The evaluation approaches and tools employed allowed for articulating beliefs around the knowledge translation activities and intended outcomes. The development of the theory of change helped in the discussion of the determinants of success for CNODES' knowledge translation activities and clarified for all team members how the activities and outputs lead to outcomes being achieved. The flexibility and iterative nature of the approach allows for updating the theory of change and the logic model as new research projects are completed and insights from early implementation efforts are used to improve the work of CNODES' knowledge translation. For example, the CNODES isotretinoin study^93^ informed a Health Canada report^125^ that called for the expansion of a more tailored knowledge translation strategy to gain further stakeholder engagement to decrease pregnancy in woman using isotretinoin. There is a need for improved communication with prescribers, pharmacists, and the public. Table 2 provides a list of additional future outcome objectives.

Working with the Nova Scotia Health Research Foundation (NSHRF) and Credentialed Evaluators ¶¶¶
Competencies for evaluators have been established in Canada. For evaluations of complex programs, there is a need for a range of competencies not generally associated with evaluation of less complex initiatives.^107^ By working with a Credentialed Evaluator (a professional designation awarded by the Canadian Evaluation Society—a professional association for evaluators in Canada),^129^ evaluation standards of Utility, Feasibility, Propriety, Accuracy, and Evaluation Accountability are applied as appropriate.^130^
 by the Canadian Evaluation Society ###
Dr Nancy Carter, lead methodologist for the evaluation, and Dorian Watts are Credentialed Evaluators by the Canadian Evaluation Society. Dr Carter also holds a PhD in Organizational Behaviour and Human Resource Management from the University of Toronto's Rothman Business School. provided researchers the support necessary for managing this highly complex evaluation.

### 5.2 Limitations

A logic model is merely a graphic representation of a program that does not always ensure its plausibility, feasibility, or success^104^ and does not consider all the structural complexities and feedback loops related to a program—in this case, the knowledge translation efforts of CNODES. An example of one feedback loop is how the Knowledge Translation Team continues to learn from each query, which then impacts knowledge translation approaches for subsequent queries in an ongoing, internal team‐based feedback loop not noted within the logic model. This limitation is especially important as CNODES has answered over 70 research queries, with insights gained each time a new project is conducted. As of January 2018, CNODES had responded to 74 queries, some of which were assessed as not feasible, and some answered by literature synthesis. CNODES generated 134 studies from 21 queries, of which 78 have been completed and the rest (56 studies) are ongoing (R.W. Platt, written communication, January 2018). A challenge in developing the theory of change was that the CNODES Knowledge Translation Team was not always aware of specific domain knowledge related to the context and human factors (eg, culture, operation, constraints, values, and norms) of Health Canada and the F/P/T drug plan program decision makers. This can result in missing important assumptions and thus potentially hindering effective communication of research results in a language that resonates with the stakeholder. The authors examined stakeholder context using mostly document review, which is limited in that one is not able to fully appreciate all aspects of the environment (eg, culture paradigms and ideology and stakeholder/researcher relationships) with this methodology. There are many approaches in evaluation to analyze stakeholder interests, needs, concerns, and other factors.^131^ Further stakeholder engagement over the course of the ongoing evaluation will allow for refinement of the theory of change and logic model as appropriate and assist in meeting the evaluation needs of stakeholders. In addition, the Knowledge Translation Team will need to revise the logic model as they gain greater understanding of the program and evidence over time.^104^

CNODES needs to continue to incorporate both formal and informal feedback from decision makers. Health Canada policy makers have expressed concerns that DSEN teams have not always been able to clearly communicate their capabilities related to responding to certain types of queries, which has led to some out of scope queries where research was not produced.^132^ Decision makers have also noted concerns related to the timelines for producing research evidence for certain queries. There are some data access delays in certain provinces. Some analyses are complex and require significant investment in time for protocol development and implementation to provide the most rigorous research evidence. For example, the studies examining the safety of antidiabetic incretin‐based drugs required the study cohort to be nested within a larger base cohort. This methodological approach, which required programming and testing between sites, allowed CNODES to study potential adverse effects among new users of these recently approved drugs while considering patients' entire history of antidiabetic drug use and avoiding potential biases associated with the study of prevalent users and left truncation. This approach also increased the generalizability of study results.^94^, ^133^ The limitation related to timelines is being addressed by the CNODES Methods and Database teams who are working on a Common Data Model approach to increase capacity across the CNODES network to be responsive to certain types of queries. CNODES' Knowledge Translation Team will need to continue to work with Health Canada and F/P/T decision makers through consultation or workshops to better understand how to be more relevant and how to communicate CNODES research results more effectively. In addition, ongoing communication between researchers and knowledge users will help manage expectations.

As unanticipated outcomes (both positive and negative) emerge through the CNODES Knowledge Translation Team evaluation, they can be incorporated into revisions of the evaluation framework, logic model, and theory of change, thereby strengthening the usefulness and relevance of the evaluation. For example, uptake of CNODES' research by media focusing on the risks of drugs may result in unintended impacts on patients such as discontinuing medication without advice from a health care professional (even when the media release clearly states to consult with their own health care provider), which can be explored as part of ongoing impact evaluation.

Another unanticipated outcome has been the “spill‐over” of CNODES' innovative analytic methods, application, and linkages of existing administrative databases which are being adopted by other organizations and researchers (eg, research method enhancements at the Manitoba Centre for Health Policy ****
Manitoba Centre for Health Policy: http://umanitoba.ca/faculties/health_sciences/medicine/units/chs/departmental_units/mchp/. and capacity development for researchers and analysts at the Saskatchewan Health Quality Council ††††
Saskatchewan Health Quality Council: http://hqc.sk.ca/.). For example, once analysts in the Manitoba Centre for Health Policy became familiar with the High‐Dimensional Propensity Scores (HDPS) approach and understood how it differed from a conventional propensity score approach, they started to employ the HDPS approach in other studies.^134^, ^135^, ^136^, ^137^ In addition, CNODES investigators and trainees have published methods papers^138^, ^139^, ^140^, ^141^, ^142^ that may be used by other researchers and decision makers and contribute to the reservoir of knowledge to be used when needed.^143^ By taking a developmental approach to the evaluation of CNODES' knowledge translation, the evaluation process can be flexible enough to allow for documenting and reporting on the impact of such unanticipated outcomes from knowledge translation activities. A limitation to date, however, has been the availability of resources to examine these outcomes.

### 5.3 Issues for the future

CNODES aims to contribute to improved drug safety and effectiveness in Canada, but there are many confounding factors which make determining the impact of any single intervention challenging. Many of the factors that influence outcomes are beyond the control of CNODES such as patients' beliefs or specific behaviors (eg, adhering to drug regimens).^144^, ^145^ In addition, government decision making related to drug therapy is likely to be informed by a variety of evidence and contextual considerations, not all of which are readily observable and easily assessed.^146^

As such, it is important to acknowledge and account for such uncertainties in complex systems which has led CNODES' Knowledge Translation Team to first focus on performance measures at the level of outputs that are within the control of the CNODES knowledge translation program. Case studies of specific queries may be helpful, while being mindful of resource implications and burden of data collection. Contribution analysis, an approach to assessing program performance developed by Mayne^100^, ^144^ to address such problems of attribution of outcomes to government policies and programs could be applied to CNODES' evaluations in the future. The evaluation framework for the CNODES knowledge translation component could be adapted for other CNODES teams. CNODES also needs to determine if it can facilitate collaborations with others who are conducting research into impact assessment (eg, CIHR and decision makers) to see if there might be opportunities to develop a common impact framework.^147^

Future research should continue to explore theories, methods, and tools that can be useful to evaluate CNODES' knowledge translation activities at the micro, meso, and macro levels. Organizational context including resources, leadership, communication, networks, and culture are key issues that need attention.^148^ CNODES can gain insights from many scientific disciplines (organizational, human factors, systems engineering, implementation science, etc.)^149^, ^150^, ^151^, ^152^ and from other industries using complex system methods such as climate change, urban science, and the airline industry.^85^ Human factor science is also a useful approach to identify facilitators and barriers to knowledge translation and improve the design of technologies, organizational structures, procedures, processes, and work systems and understand both internal and external factors (eg, political and economic factors).^124^, ^153^, ^154^

## 6 CONCLUSION

The use of a developmental evaluation approach and an evaluation framework for assessing the impact of the knowledge translation component for CNODES will provide guidance as CNODES strives to learn from its ongoing research projects, adapt its policies and approaches, and document its research impact. Other pharmacoepidemiologic researchers could adapt and expand on the framework for their own impact assessments.

## ETHICS STATEMENT

This research did not require ethics approval as it was based on review of published and publicly available literature.

## Supporting information

Data S1. Supporting information
