# Supplementary material for: Building a framework for the evaluation of knowledge translation for the Canadian Network for Observational Drug Effect Studies
Source: Pharmacoepidemiol Drug Saf. 2019 Feb 20;29(Suppl 1):8–25. doi: 10.1002/pds.4738 (PMC6972643; doi:10.1002/pds.4738)
Supplement: Supplementary file 1 — Data S1. Supporting information [file PDS-29-8-s001.docx]

# Supplementary Material

# **Appendix S1a.** Health Canada branches and agencies, including CIHR and its relationship with CNODES

Adapted from <http://www.hc-sc.gc.ca/ahc-asc/branch-dirgen/index-eng.php> and <http://www.cihr-irsc.gc.ca/e/45799.html>.

**Appendix S1b.** The Health Products and Food Branch (HPFB) selected Directorates and Offices

| **Organization** | **Mandate** | **Activities** | **Offices and Bureaus** |
| --- | --- | --- | --- |
| Marketed Health Products Directorate (MHPD)  <http://www.hc-sc.gc.ca/ahc-asc/branch-dirgen/hpfb-dgpsa/mhpd-dpsc/index-eng.php> | *“…works to assure that Health Product and Food Branch (HPFB) programs take a consistent approach to post-approval safety surveillance, assessment of signals and safety trends and risk communications concerning all regulated marketed health products.”* | Monitors and collects adverse reaction and medication incident data; reviews and analyzes marketed health product safety data. MHPD conducts benefit-risk assessments of marketed health products and communicates product-related risk to health care professionals and the public. For example, MHPD developed the Medeffect program to provide centralized access to relevant and reliable health product safety information as it becomes available, in an easy to find, easy to remember location. This includes access to Health Canada's advisories, warnings, and recalls; the Canadian Adverse Reaction Newsletter (CARN); and the Canadian Adverse Drug Reaction Monitoring Program (CADRMP) Online Query and Data Extract. MHPD also oversees the advertising regulatory requirement of health products and provides policies to effectively regulate marketed health products. | - Director General’s Office - Bureau of Strategic Initiatives and Planning - Marketed Biologicals, Biotechnology and Natural Health Products Bureau - Marketed Health Products Safety and Effectiveness Information Bureau - Marketed Pharmaceuticals and Medical Devices Bureau - Therapeutic Effectiveness and Policy Bureau |
| Policy, Planning and International Affairs Directorate (PPIAD)  <http://www.hc-sc.gc.ca/ahc-asc/branch-dirgen/hpfb-dgpsa/ppiad-dppai/index-eng.php> | N/A | Provides leadership on implementing HPFB’s strategic planning process and assisting the Branch Executive Committee in making it the basis for HPFB decision-making on resource allocation, performance measurement and reporting, and communicating with government and stakeholders on strategic directions and performance plans and results. PPIAD supports the development of Branch policy and planning capacity and provides high quality and timely advice for the Assistant Deputy Minister and Branch Executive Committee on strategic policy and planning issues. | N/A |
| Therapeutic Products Directorate (TPD) <http://www.hc-sc.gc.ca/ahc-asc/branch-dirgen/hpfb-dgpsa/tpd-dpt/index-eng.php> | *“… federal authority that regulates pharmaceutical drugs and medical devices for human use.”* | TPD regulates pharmaceutical drugs and medical devices for human use. Prior to being given market authorization, a manufacturer must present substantive scientific evidence of a product's safety, efficacy, and quality as required by the Food and Drugs Act and Regulations*.* | There are 15 bureaus; those of note here include:   - Bureau of Policy, Science and International Programs - Office of Risk Management - Regulatory Affairs |

**Appendix S2.** CNODES’ key knowledge users and stakeholders

1. **PRIMARY KNOWLEDGE USERS**

| **Audience** | **Message** | **Product / Strategy** |
| --- | --- | --- |
| **Canadian Institutes of Health Research (CIHR)**   - Drug Safety and Effectiveness Network (DSEN) | - Use/benefits of administrative data - Integrated knowledge translation, leading to the production of relevant, applicable research - The success of cross-country collaboration | - Presentations at DSEN network meetings and briefings to CIHR’s DSEN office - Involve at query submission and refinement, including project timelines, preliminary results/reports, and final results presentation |
| **Health Canada**   - Therapeutic Products Directorate (TPD) - Marketed Health Products Directorate (MHPD) | - Decrease uncertainty in estimation of benefits and risks - Provide results at provincial node and UK and US regulatory agencies, where relevant - Highlight breadth and depth of available data and analytical approaches and benefits of knowledge gained - Highlight uniqueness of approach in the Canadian context, the power of administrative data already collected, and a strong case for the collection of more administrative data (e.g. more variables, more sources, more regions) | - Involve at query submission and refinement, including project timelines, preliminary results/reports, and final results presentation - Share PowerPoint slide decks of results with Query Submitters and provide the opportunity to dialogue with clinicians and methodologists - CNODES’ participation in all DSEN/Health Canada/F/P/T meetings to report on CNODES |
| **Federal / Provincial / Territorial (F/P/T) Pharmacare Programs**   - Provincial/Territorial Listing Review - Federal Public Drug Benefit Programs | - The capabilities and availability of CNODES to provide information useful for a number of pharmacare functions and the encouragement to provide further queries - Benefit and/or risk quantification for specific provincial nodes - Drug utilization by provincial or F/P/T pharmacare programs | - Involve in query process, including project timelines, scientific protocols, preliminary results/reports, and final results presentation - Participation in organized meetings with Health Canada and F/P/T decision maker participants to report on CNODES - Articles, website, presentations - F/P/T pharmacare programs may provide direct input into the conduct of the project to answer the query by participating at specific stages in the project team |

1. **OTHER STAKEHOLDERS**

| **Government and Selected National or Pan-Canadian Organizations**   - Parliament - Senate - Institut national d’excellence en santé et en services sociaux (INESSS) - Canadian Agency for Drugs and Technologies in Health (CADTH) - Common Drug Review (CDR) (including Canadian Drug Expert Committee (CDEC)) - Canadian Patient Safety Institute (CPSI) - Canadian Institute for Health Information (CIHI) - National Prescription Drug Utilization Information System Database (NPDUIS) (developed in consultation with the Patented Medicine Prices Review Board (PMPRB)) |
| --- |
| **Selected Public Sector and Healthcare Professionals**   - CNODES and their networks (including DSEN-funded collaborating teams, CNODES Steering Committee members) - Health care regulatory (College of Physicians and Surgeons, College of Pharmacy, nursing regulatory bodies) and professional bodies (e.g. CMA, CPhA, CNA) - Health care delivery organizations (including health care providers and managers practicing in primary care, continuing care, long-term care, and acute care settings) - Federal/Provincial Public Health Authorities/Departments |
| **Researchers and Trainees**   - Junior/new-to-field faculty/clinician scientists - Trainees - CNODES node and affiliated researchers - Research analysts and other health care providers - Academic educators (Faculties/Schools of Nursing, Medicine, Pharmacy) |
| **International Researchers, Industry, Media, Voluntary Health Sector, Patient Groups**   - International researchers and health care regulatory and quality improvement organizations (including Sentinel and PROTECT, MedicineWise) - Industry-Pharmaceutical, Publishing, Legal - Media (including social media) - Citizens / public / patients / caregivers - Voluntary and not-for-profit health organizations (e.g. Heart and Stroke, CCS, ISMP Canada, CSHP) |

**Appendix S3.** Definitions of select terms pertaining to research impact assessment and knowledge translation (arranged alphabetically)

| **Altmetrics** | Altmetrics is the study and use of non-traditional research impact measures based on engagement with research outputs in web-based sources, particularly social media. Altmetrics are an alternative and complementary means to more traditional methods (e.g. journal impact factor, h-index, citation metrics) for measuring research uptake. These metrics include mentions on blogs, shares and “likes” on social media (Twitter, Facebook), and posts using reference managers.^1^ |
| --- | --- |
| **Citation metrics** | Citation metrics (or bibliometrics) is an approach for measuring research impact based on citation counts (e.g. the number of times an article is cited). Citation metrics may be used as indicators of scholarly impact (e.g. academic rank, tenure and promotion, honours and awards, grant applications, etc.), but consensus is lacking on the most accurate and rigorous way to collect this data.^2^ |
| **Complexity** | Complexity refers to a situation in which many interacting and interdependent elements exist, within a dynamic environment, with a high degree of uncertainty and limited central control.^3^ |
| **Contribution analysis** | Contribution analysis is an evaluation method for identifying and assessing the extent to which a given program or intervention has had an impact (i.e. its contribution) on observed results.^4^ |
| **Developmental evaluation** | Developmental evaluation informs and supports innovative and adaptive development in complex dynamic environments.^3^ The purpose is to generate feedback to inform the ongoing development and/or implementation of an intervention or program. This approach is particularly useful for evaluating complex initiatives, with multiple stakeholders and evaluation needs, in a rapidly changing environment.^5^ (Related terms: *real time, emergent, action,* and *adaptive evaluation*) |
| **Distributed network** | A distributed network of data centres allows for each centres’ data to be housed and analyzed locally, thus respecting each jurisdiction’s specific privacy and data access regulations and approval processes.^6^ |
| **End of grant knowledge translation** | End of grant (or end of project) knowledge translation refers to any activity aimed at communicating or applying research results. Best practice involves defining a clear goal and tailoring both the message and mode of delivery to each intended audience.^7^ |
| **Indicator** | An indicator is a specific, measureable, attainable, realistic, and timely means of reflecting change and measuring achievement connected with a program or intervention.^8^ |
| **Integrated knowledge translation** | Integrated knowledge translation, or the co-production of knowledge, is defined as an ongoing relationship between researchers and research users for the purpose of collaboratively engaging in a mutually beneficial research project or programme of research to support decision making.^9^ |
| **Journal impact factor** | Academic journals are ranked according to their impact factor. A journal impact factor is based on the number of citations its articles received in the current year divided by its total number of articles during the preceding two years.^10^ |
| **Knowledge translation** | Knowledge translation is defined as a dynamic and iterative process, involving interactions between researchers and research users, that includes synthesis, dissemination, exchange, and ethically sound application of knowledge to improve health and strengthen the health care system.^7^ |
| **Logic model** | A logic model is a results chain that describes a program’s main activities (sometimes called components, services, or interventions) and their expected outputs and outcomes.^11^ |
| **Nonlinearity** | Nonlinearity is a concept in complexity that refers to how small actions can lead to disproportionately large reactions. Nonlinearity is expected to be high when levels of uncertainty are also high.^12^ |
| **Output** | An output is a product resulting from a specific program or intervention activity (e.g. an article).^11^ |
| **Outcome** | An outcome is the impact or result of a given output. This may refer to short- (e.g. increased knowledge of research results), intermediate- (e.g. increased use of research results in practice), or long-term (e.g. improved health) results.^11^ |
| **Pharmacoepidemiology** | Pharmacoepidemiology is the application of epidemiological methods to pharmacological issues; it is the study of the uses and effects of drugs in large, well-defined populations.^13^ |
| **Reach** | Reach refers to the target (e.g. individuals, organizations, other stakeholders) that a program or organization is trying to influence.^14^ |
| **Receptor capacity** | Receptor capacity refers to the ability and willingness of an intended audience to access, understand, and use information.^15^ |
| **Research impact assessment** | Research impact refers to the assessment of research outputs and their influence as an evaluation of the research occurring within an institution.^16^ |
| **Theory of change** | A theory of change is a visual representation of a program or intervention that considers how activities are expected to achieve given outcomes by providing theoretical causal links between program components, outputs, and outcomes, with assumptions that must be true for these links to be accurate.^11,17^ |
| **Utilization-focused evaluation** | A utilization-focused approach considers stakeholders’ intended uses of the evaluation results from the outset. It is a key part of developmental evaluation.^3^ |

**Appendix S4.** List of abbreviations appearing in the text, footnotes, tables, figures, and appendices (arranged alphabetically)

| **CADRMP** | Canadian Adverse Drug Reaction Monitoring Program |
| --- | --- |
| **CADTH** | Canadian Agency for Drugs and Technologies in Health |
| **CAHS** | Canadian Academy of Health Sciences |
| **CAN-AIM** | Canadian Network for Advanced Interdisciplinary Methods for Comparative Effectiveness Research |
| **CARN** | Canadian Adverse Reaction Newsletter |
| **CDEC** | Canadian Drug Expert Committee |
| **CDR** | Common Drug Review |
| **CIHI** | Canadian Institute for Health Information |
| **CIHR** | Canadian Institutes of Health Research |
| **CMA** | Canadian Medical Association |
| **CNA** | Canadian Nurses Association |
| **CCS** | Canadian Cancer Society |
| **CNODES** | Canadian Network for Observational Drug Effect Studies |
| **CPhA** | Canadian Pharmacists Association |
| **CPSI** | Canadian Patient Safety Institute |
| **CSHP** | Canadian Society of Hospital Pharmacists |
| **DSEN** | Drug Safety and Effectiveness Network |
| **DSRU** | Drug Safety Research Unit |
| **EFPIA** | European Federation of Pharmaceutical Industries and Associations |
| **EMA** | European Medicines Agency |
| **ENCePP** | European Network of Centres for Pharmacoepidemiology and Pharmacovigilance |
| **EU-ADR** | Exploring and Understanding Adverse Drug Reactions by Integrative Mining of Clinical Records and Biomedical Knowledge |
| **FDA** | Food and Drug Administration |
| **F/P/T** | Federal / Provincial / Territorial |
| **FNIH** | Foundation for the National Institutes of Health |
| **HPFB** | Health Products and Food Branch |
| **GSK** | GlaxoSmithKline |
| **ICH** | International Conference on Harmonization of Technical Requirements for Registration of Pharmaceuticals for Human Use |
| **ICMRA** | International Coalition of Medicines Regulatory Authorities |
| **IMEDS** | Innovation in Medical Evidence Development and Surveillance |
| **INESSS** | Institut national d’excellence en santé et an services sociaux |
| **ISMP** | Institute for Safe Medication Practices |
| **ISPE** | International Society for Pharmacoepidemiology |
| **ISRIA** | International School on Research Impact Assessment |
| **KT** | Knowledge translation |
| **MAGIC** | Methods and Applications for Indirect Comparisons |
| **MATRICE** | The Integration of Content Management Information on the Territory of Patients with Complex Diseases or with Chronic Conditions |
| **MHPD** | Marketed Health Products Directorate |
| **NPDUIS** | National Prescription Drug Utilization Information System Database |
| **NSHRF** | Nova Scotia Health Research Foundation |
| **OMOP** | Observational Medical Outcomes Partnerships |
| **OPI** | Office of Pediatric Initiatives |
| **OCS** | Office of Controlled Substances |
| **PMPRB** | Patented Medicine Prices Review Board |
| **PPIAD** | Policy, Planning and International Affairs Directorate |
| **PPX** | Performance and Planning Exchange Symposium |
| **PRAC** | Pharmacovigilance Risk Assessment Committee |
| **PREVENT** | Pharmacogenomics of Adverse Events National Team |
| **PROTECT** | Pharmacoepidemiological Research on Outcomes of Therapeutics by a European Consortium |
| **RUF** | Reagan-Udall Foundation |
| **SALUS** | Scalable, Standard based Interoperability Framework for Sustainable Proactive Post Market Safety Studies |
| **SEARCH** | DSEN Active Surveillance and Evaluation of Adverse Reactions in Canadian Healthcare |
| **TPD** | Therapeutic Products Directorate |
| **VRMM** | Vigilance and Risk Management of Medicines |

# References for Supplementary Material

1. Priem J, Groth P, Taraborelli D. The altmetrics collection. *PLoS One* 2012;**7**(11):e48753.
2. Rothfus M, Sketris IS, Traynor R, *et al*. Measuring knowledge translation uptake using citation metrics: A case study of a pan-Canadian network of pharmacoepidemiology researchers. *Science & Technology Libraries* 2016;**35**(3):228-240.
3. Patton MQ. Developmental Evaluation: Applying Complexity Concepts to Enhance Innovation and Use. New York, NY: Guilford Press; 2011.
4. Mayne J. Contribution analysis: An approach to exploring cause and effect. *ILAC* 2008;Brief 16.
5. Dozois E, Langlois M, Blanchet-Cohen N. DE 201: A Practitioner's Guide to Developmental Evaluation. Montreal, QC: The J.W. McConnell Family Foundation and the International Institute for Child Rights and Development; 2010.
6. Suissa S, Henry D, Caetano P, *et al*. CNODES: The Canadian Network for Observational Drug Effect Studies. *Open Medicine* 2012;**6**(4):e134.
7. Canadian Institutes of Health Research. Guide to knowledge translation planning at CIHR: Integrated and end-of-grant approaches. 2015; Available at: <http://www.cihr-irsc.gc.ca/e/45321.html>. Accessed September 25, 2017.
8. Organisation for Economic Co-Operation and Development. DAC Guidelines and Reference Series: Quality Standards for Development Evaluation. 2010; Available at: <http://www.oecd.org/dac/evaluation/qualitystandardsfordevelopmentevaluation.htm>. Accessed September 25, 2017.
9. Gagliardi AR, Dobrow MJ. Identifying the conditions needed for integrated knowledge translation (IKT) in health care organizations: qualitative interviews with researchers and research users. *BMC Health Serv Res* 2016;**16**:256-264.
10. Garfield E. The history and meaning of the journal impact factor. *JAMA* 2006;**295**(1):90-93.
11. Knowlton LW, Phillips CC. Chapter 8: Theory of Change and Program Logic Models. In: Grinnell RM, Gabor PA, Unrau YA, editors. Program Evaluation for Social Workers: Foundations of Evidence-Based Programs. 7th ed. New York, NY: Oxford University Press; 2016. p. 165-190.
12. Ofek Y. The missing linkage in evaluating networks: a model for matching evaluation approaches to system dynamics and complexity. *Public Perform Manag* 2015;**38**(4):607-631.
13. International Society for Pharmacoepidemiology. About Pharmacoepidemiology. 2017; Available at: <https://www.pharmacoepi.org/about/about.cfm>. Accessed September 25, 2017.
14. Montague S, Porteous NL. The case for including reach as a key element of program theory. *Eval Program Plann* 2013;**36**:177-183.
15. Denis J, Lomas J, Stipich N. Creating receptor capacity for research in the health system: The Executive Training for Research Application (EXTRA) program in Canada. *J Health Serv Res Policy* 2008;**13**(Suppl 1).
16. Penfield T, Baker MJ, Scoble R, *et al*. Assessment, evaluations, and definitions of research impact: A review. *Res Eval* 2014;**23**(1):21-32.
17. Rogers P. Methodological Briefs: Theory of Change. Impact Evaluation No. 2. Florence, Italy: UNICEF Office of Research; 2014.
